# Note Respecting the Conduct of Dr. Wilson Philip.

**Published:** 1820-05

**Authors:** W. Hutchinson

**Affiliations:** Sackville-street


### *Note respecting the Conduct of* Dr. Wilson Philip. By Mr. Hutchinson.

IN the Quarterly Medico-Chirurgical Journal for April last, Dr. Philip has thought proper to publish the same remarks on some passages in my Proëmium as appeared in this Journal for March, notwithstanding the manner in which I had replied to them: how far I refuted the principal points in Dr. Philip's remarks, must be determined by others. Dr. Philip has also, without including my Reply, added some remarks on that Reply, which, according to the common rules of candid controversy, should have comprised a refutation of my objections to Dr. Philip's charges in his former remarks, or the publication of those charges should not have been subsequently made.* Instead of which, he has added mis-statements of such a kind, that I should not have thought them proper for my notice, had I not been previously led into this controversy. I shall point them out in the order in which they occur. I may previously mention, that Dr. Philip persists in calling me a *reviewer*, as the writer of that Proëmium; notwithstanding my remarks on this point in my Reply, and the evidence in the character of the Proëmium itself. He has heard, I suppose, candid and liberal men express themselves in a particular manner respecting the duty of a reviewer; and he thinks thus to prejudice the public against me, and give to the trivial errors I committed as an *historian* a more serious character.

He adds, that, in my Reply, I have but little amended my former statements, because I call Legallois "the executor of those experiments which most clearly prove that the action of the heart continues after the destruction of the spinal marrow;" whereas, he says, "All those who are acquainted with the work of M. Legallois know that his chief object was to prove, that the action of the heart does *not* continue after the destruction of the spinal marrow." It is difficult to say which Dr. Philip displays here in the most glaring manner, his folly, or his artfulness in making mis-statements. Every rational man will say to Dr. Philip, "It signifies little to me what Legallois' *chief object was to prove*, but what *has he proved*." Now, this is just what I plainly expressed in my Proëmium. I say there, "It may not be devoid of utility to show how the very experiments from which these arguments have been deduced (that the heart depends on the spinal marrow for its motive influence), tend to support the side of the question they are considered to oppose; and that they do this in a very powerful manner, may be easily proved." (*Proëmium*, p. xxi.)

Dr. Philip objects to my calling him "the repeater of the greater part of Legallois' experiments:" he says, "In the whole of my

* The reader should be informed, that Dr. Philip did not offer his last remarks for insertion in this Journal, where my Reply had appeared, although he afterwards sent a communication on another subject; but published them in another Journal, for the perusal of many persons who may be supposed to have seen neither the Reply nor the Proëmium.

Treatise I relate the repetition of but *one* of this author's experiments." What, I ask any person who has read his *Inquiry*, are all Dr. Philip's experiments on the influence of the destruction of the spinal marrow on the action of the heart, but repetitions of those of Legallois?

Dr. Philip, as an effort to obviate my reason for using the term *reservoir* as designative of a notion of his, says, I quote from his Treatise "observations which apply to the *nerves in general*, as a proof that I consider the *ganglian system* a reservoir of nervous influence!" I need only request the reader to turn to the passages I have quoted, if he does not recollect that the term "THE GANGLIAN SYSTEM" is used by Dr. Philip in one of the cited passages.

Dr. Philip commences his next paragraph with, "I do not wish to proceed farther;" yet this evidence of consummate self-conceit is followed by the citation of *half a sentence* from my Reply, because I in that sentence confessed the two trivial errors I began my reply with acknowledging; and then Dr. Philip artfully adds, "If he recur to my observations, he will find that it was applied not to one, but many errors, which I had enumerated, as appears from the first words of the sentence, "*all this*." The reader will not forget that Dr. Philip acts thus after the publication of my Reply; and I repeat, on this point, as well as in respect to his last remarks in general, it is new in the history of controversy for a man to publish charges subsequently to objections to them which he has shown himself unable to refute.

With respect to the two errors in question, I add, that every person of extensive reading in medical literature knows that there is hardly an author who has noticed to a great extent the opinions of others, that has not in some instances erred in what he has attributed to them. I need not therefore, I am confident, fear, that the conduct of Dr. Philip on this occasion will make of such serious importance in me, what is regarded in others as an error necessarily venial, for it is one which no person can hope to avoid in multitudinous references to the history of so comprehensive a subject as medicine.*

W. HUTCHINSON.

*Sackville-street; April 28th,* 1820.

---

* The above remarks are published in this Journal, 1°, because it is necessary to *pay the expense of their publication* in the Journal in which Dr. Philip published the papers in question, (the editor affixing them to his Journal as *an appendix of additional matter;*) and the expense of printing a single leaf amounts to a considerable sum, very disproportionate to that for a whole sheet; a sum I do not choose to spend on this occasion: because, 2°, my chief wish is to vindicate myself to the readers of this Journal, and those persons who will not form their judgment on the subject until they have examined every thing relating to it.

